# Plant Systems Biology in 2022 and Beyond

**DOI:** 10.3390/ijms23084159

**Published:** 2022-04-09

**Authors:** Tsanko Gechev, Veselin Petrov

**Affiliations:** 1 Center of Plant Systems Biology and Biotechnology, 4000 Plovdiv, Bulgaria; vpetrov@plantgene.eu; 2 Department of Plant Physiology and Molecular Biology, University of Plovdiv, 4000 Plovdiv, Bulgaria; 3 Department of Plant Physiology, Biochemistry and Genetics, Agricultural University—Plovdiv, 4000 Plovdiv, Bulgaria

Plants have remarkable plasticity due to their vast genetic potential which interacts with many external factors and developmental signals to govern development and adaptation to changing environments. Systems biology unravels this genetic potential and the related intricate molecular interactions using a holistic approach, revealing how genes, proteins, and metabolites function together to realize this plasticity under specific conditions. Our knowledge about these fundamental molecular mechanisms can be exploited not only to prepare more resilient crops but also to produce particular molecules (proteins, metabolites) with applications in medicine and cosmetics in a sustainable biotechnological way.

The Special Issue “Selected Papers from the International Conference on Plant Systems Biology and Biotechnology 2020” signifies various topics covered by the conference, including plant interactions with the environment (abiotic, biotic, and oxidative stresses), plant genetic resources, as well as analysis and production of plant-derived molecules with potential applications in medicine and cosmetics. We believe the Special Issue will be of interest to a diverse readership of plant biologists.

Drought is the predominant abiotic stress that affects both plants in natural ecosystems and crops. Rasul et al. (2021) have demonstrated that a commercially available extract produced from the brown alga *Ascophyllum nodosum* protects *Arabidopsis thaliana* from drought by activating its natural defense system [1]. The authors showed that Arabidopsis pre-treated with the *A. nodosum* extract Super Fifty (SF) accumulates less reactive oxygen species (ROS) and exhibits less damage than untreated plants exposed directly to drought. Moreover, SF-treated plants did not manifest the growth inhibition characteristic for the controls subjected to this stress. Comprehensive transcriptome analysis by RNA-seq revealed induction of many ROS marker genes in the untreated plants, consistent with the observed oxidative stress, whereas no such induction was detected in the SF-treated group. Expression of the *RESPONSE TO DESSICATION 26 (RD26)* gene, encoding a transcription factor known to be a stress-responsive negative growth regulator, was upregulated in the drought stressed plants but repressed by the SF treatment. Consistent with this, evaluation of the gene expression of the cell cycle marker gene *HISTONE H4* *(HIS4)* by in situ hybridization at the shoot apical meristem (SAM) demonstrated that this SAM marker is completely turned off in the drought stressed plants while SF maintains its expression even under water limiting conditions. These findings provide a mechanistic understanding how drought represses growth at the molecular level and how SF can prevent both drought stress damage and growth inhibition. The authors also implicate abscisic acid (ABA) signaling and the SnRK2 pathway in the mechanisms of SF-induced drought mitigation. Collectively, the data show that SF can be used as an effective ecologically friendly molecular priming technology to protect plants from drought stress.

Oxidative stress, manifested as increased levels of ROS, often accompanied by macromolecular and cellular damage, is a consequence of most abiotic and biotic stresses [2]. ROS are also important signaling molecules that control numerous developmental processes and stress responses [3]. Maintaining the ROS balance by the antioxidant system is therefore critical both for proper plant development and for stress acclimation. Lyal et al. (2020) have performed a cross-kingdom comparison of the ROS network in order to identify gene families that are expanding or contracting in the genomes of 37 plants and 28 non-plant species (protists, fungi, and animals) [4]. Particular focus was given on the extremophile species: organisms that can survive extreme environments, such as severe drought/desiccation, high salinity, extreme temperatures. While catalases, superoxide dismutases, glutathione reductases, peroxidases and glutathione peroxidase/peroxiredoxins are present in all kingdoms, ascorbate peroxidases, dehydroascorbate reductases and monodehydroascorbate reductases appear to be specific for plants [4]. Tardigrades and rotifers do show ROS gene expansions that could be related to their extreme lifestyles, although a high rate of lineage-specific horizontal gene transfer events, coupled with recent tetraploidy in rotifers, could explain this observation. Most of the extremophile species from the animal and plant kingdoms, however, do not show any drastic expansion of antioxidant gene families, suggesting that the basal eukaryotic ROS scavenging systems are sufficient to maintain ROS homeostasis even under the most extreme conditions and that prompt regulation of gene expression is probably more important for the adaptation to such environments. 

In addition to abiotic stresses, biotic stresses pose further threat to plant health and, in case of agriculturally important plants, to crop yield. The water mold *Phytophthora* *cinnamomi* is one of the most invasive pathogens which can attack and destruct many wild species and crops. Its wide host range is remarkable, but little is known about the mechanisms of this broad invasiveness. The team of Kerchev et al. (2020) have studied the molecular interactions of *P. cinnamomi* with *Castanea sativa* in order to understand how this pathogen spreads and design ways to control it [5]. Using stem infection as a model system, the authors performed comprehensive proteome and metabolome analyses combined with targeted hormonal analysis to identify proteins, metabolites, and pathways that are affected during the plant-pathogen interaction and may be responsible for the transmission of the pathogen. In this model, samples were taken from both the proximal stem zone near the *P. cinnamomi* inoculation site and the distal zone away from it. The pathogen was growing only in the proximal zone, as evidenced by the observed necrotic areas and confirmed by the presence of *P. cinnamomi* proteins in the proximal but not the distal area. Both zones, especially the proximal one, revealed accumulation of proteins associated with biotic and abiotic stress responses. Interestingly, proteins related to hydrogen peroxide metabolic processes and heat shock responses were the two most decreasing in abundance categories in the proximal zone [5]. Hence, *P. cinnamomi* is able to repress the oxidative burst necessary to mount proper defense response and the induction of heat shock proteins that are known to protect from biotic and abiotic stresses. This observation provides a mechanistic understanding of *P. cinnamomi* pathogenicity. The authors note also activation of salicylic acid and jasmonic acid signaling, which play a role in *P. cinnamomi-C. sativa* interactions by modulating several biochemical processes and stress responses.

In another study dedicated to biotic stress, Mishev et al. (2021) investigated the plant hormonome in the tripartite system of tobacco (*Nicotiana tabacum*)—the arbuscular mycorrhizal (AM) fungus *Rhizophagus irregularis*—and the parasite plant broomrape (*Phelipanche* spp.) [6]. Plant roots release chemical signals that stimulate the development of beneficial AM fungi but some parasitic plants utilize a few of these signals for their own germination and growth. The hormone content of roots and exudates from plants colonized with *R. irregularis* and infested with *Phelipanche* spp. resembled the exudates from infested plants without the AM fungus, whereas the exudates of non-infested plants colonized with *R. irregularis* were rather different. In particular, both roots and exudates from parasite infested plants not colonized with *R. irregularis*, and exudates from parasite infested plants colonized with *R. irregularis* showed elevated levels of ABA [6]. In contrast, tobacco plants that were not infested with *Phelipanche* spp. did not have higher ABA levels, neither in the roots nor in the exudates. While the auxin indole-3-acetic acid and cytokinine levels increased in the roots of all cases, these hormones decreased in exudates from the non-infested sample. In these plants, the AM fungus *R. irregularis* was able to colonize the roots much better, suggesting that the parasite plant *Phelipanche* spp. has a high impact on the rhizosphere interactions of its host, since it is able to suppress the AM colonization [6]

Genetic diversity plays a major role in plant responses to the environment and determines important agronomical traits and yield in crops. Nankar and Prat (2021) have studied the genetic diversity of six blue maize landraces located in North America [7]. Blue maize is highly valued for its nutritional properties and the content of anthocyanins (cyanidin 3-glucoside, pelargonidin, and peonidin 3-glucoside) [8]. Using tunable genotyping-by-sequencing (tGBS™), they identified 81,038 high quality SNPs that determine genetic relatedness of these maize landraces [7]. The genotypic analysis revealed that one of the varieties—‘Ohio Blue’, characteristic for the Corn Belt, differs significantly from southwestern landraces. In turn, it was confirmed that the southwestern landraces are closely related, despite the observed diversity of phenotypes achieved through continuous active selection. In parallel, the authors evaluated 45 morphological traits, including ear height, kernels per year, number of ears per plant, weight of cob, and grain yield, as well as biochemical traits such as total fatty acids, protein, starch, oil and anthocyanin content [7]. Kernel traits appeared to have the highest variability, while plant traits (plant height, number of tillers, secondary branches, nodes and internodes, etc.) had the lowest. Overall, this study provides valuable insights about the genetic diversity and the most important agronomical properties of these unique land races of blue maize.

Secondary metabolites with nutritional and health promoting properties can be found in both crops and wild plants. Caffeic acid (CA) and chlorogenic acid (CGA), major secondary metabolites in coffee and tea, are known to inhibit ROS formation and lipid oxidation [9,10]. CGA is reported to have anti-inflammatory, anti-hyperglycemic, hepatoprotective, and cardioprotective effects [9,11]. The team of M. Georgiev has studied the anti-obesity potential of CA and CGA in human adipocytes [12]. Using a human preadipocyte cell strain derived from an infant patient with the rare Simpson–Golabi–Behmel syndrome (SGBS) as an in vitro model system, the authors showed that CA and CGA synergistically promote lypolysis [12]. Furthermore, the authors demonstrated that CA and CGA stimulate the browning process: transformation of white adipose tissues, which serve mainly as depot for energy storage, into brown/beige adipose tissues, which are responsible for thermogenesis and energy expenditure. The energy-dissipating abilities of beige/brown adipose tissues may be beneficial for metabolic disorders such as obesity and type 2 diabetes. The authors showed that CA and CGA-induced browning in SGBS preadipocytes is associated with downregulation of lipogenic markers such as *ACC*, *FASN*, and *SREBP1*, downregulation of genes for white adipogenic differentiation such as *ADIPOQ*, *CEBPA*, and *FABP4*, and induction of brown/beige markers (*CD137*, *CIDEA*, *PDK4*, *PGC1A*, *PPARA*, *UCP1*) [12]. Furthermore, molecular docking analysis revealed the ability of CA/CGA to interact with AMP-activated protein kinase (AMPK) and peroxisome proliferator-activated receptor gamma co-activator 1 alpha (PPAR), key proteins that regulate energy homeostasis [12]. Altogether, this study concluded that the AMPK signaling pathway is the most prominent for the anti-adipogenic effects of CA/CGA and that the browning is mediated through AMPK and PPARα/-γ dependent pathways. 

Myconoside and calceolarioside are secondary metabolites from *Haberlea rhodopensis*, a desiccation tolerant plant which accumulates these metabolites in high amounts upon dehydration. Myconoside was previously reported to protect human skin from UV radiation and oxidative damage, to stimulate the synthesis of elastin and collagen, to increase skin elasticity and enhance skin radiance [13,14]. Hence, medications with topical application of myconoside extracts were suggested as anti-aging treatment of human skin. Amirova et al. (2021) went further and investigated the effect of myconoside and calceolarioside on the expression of nuclear factor erythroid 2 p45-related factor 2 (*Nrf2*) gene in bone marrow neutrophils [15]. The transcription factor Nrf2 is a key regulator of ROS homeostasis and its activity is essential for proper expression of antioxidant enzymes and counteracting oxidative stress [16]. As *H. rhodopensis* is a protected species, the authors developed a system to grow this plant in a sustainable biotechnological way. Moreover, the in vitro cultivated *H. rhodopensis* accumulated myconoside and calceolarioside to much higher levels than the Haberlea plants grown in nature. Furthermore, the authors show that myconoside and calceolarioside rich extracts of *H. rhodopensis* activate Nrf2 both alone and together in a synergistic manner [15]. 

The articles collected in the Special Issue “Selected Papers from the International Conference on Plant Systems Biology and Biotechnology 2020” highlight various aspects of the molecular mechanisms behind plant responses to environment, the importance of genetic diversity for phenotype and crop yield, and accentuate several secondary metabolites with potential for therapeutic use in medicine. Altogether, they show the power of systems biology and biotechnology to address diverse fundamental questions related to plant development and stress responses as well as to find solutions for enhancement of crop yield and biotechnological production of plant derived medications. These directions in plant biology are currently developing very fast and the scientific output will continue to grow in the coming years. Thus, to provide a suitable platform for knowledge-sharing, networking and results dissemination, our goal will be to organize the International Conference on Plant Systems Biology and Biotechnology on a biennial basis. 

## Data Availability

Not applicable.

## Abbreviations

ABA	Abscicic Acid
AM	Arbuscular Mycorrhiza
AMPK	AMP-activated protein kinase
CA	Caffeic acid
CGA	Chlorogenic acid
CK	Cytokinin
ROS	Reactive Oxygen Species
SAM	Shoot Apical Meristem
SF	Super Fifty
tGBS	tunable Genotyping-by-Sequencing
